# Impact of Bupivacaine on malignant proliferation, apoptosis and autophagy of human colorectal cancer SW480 cells through regulating NF-κB signaling path

**DOI:** 10.1080/21655979.2021.1937911

**Published:** 2021-06-21

**Authors:** Bingwu Liu, Xinfeng Yan, Zuojia Hou, Lei Zhang, Duwen Zhang

**Affiliations:** a Department of Anesthesiology, The Second Children and Women’s Healthcare of Jinan City, Jinan City, Shandong Province, China; b Department of Anesthesiology, Laiwu People’s Hospital of Jinan City, Jinan City, Shandong Province, China; c Department of Anesthesiology, Laiwu Iron and Steel Group Laiwu Mining Co., Ltd. Staff Hospital, Jinan City, Shandong Province, China; d Department of Anesthesiology, Guizhou Provincial People’s Hospital, Guiyang City, Guizhou Province, China

**Keywords:** Bupivacaine, colorectal cancer, nf-κB, apoptosis, autophagy

## Abstract

To probe into the impact of Bupivacaine on colorectal cancer (CRC) proliferation, apoptosis, and autophagy through regulating the NF-κB signaling pathway. Our work treated CRC cells with Bupivacaine, detected cell vitality through MTT assay, apoptosis through flow cytometry, cell migration through wound healing assay, NF-κB activity through immunofluorescence, inflammatory factor level, including TNF-α, IL-1β as well as IL-6 through ESLIA, apoptosis factor mRNA expression, including Bcl-2, Bax and caspase-3q through qRT-PCR, and protein expression linking with NF-κB signaling pathway as well as autophagy-related proteins via western blot. In in vivo experiments, we explored the impact of Bupivacaine on tumor volume, tumor and NF-κB expression. The results showed that 1 mM Bupivacaine was available to signally inhibit CRC cell vitality, promoted apoptosis rate and apoptosis gene expression, like Bax, and caspase-3, inhibited Bcl-2 expression, inhibited cancer cell migration, promoted autophagy-related protein LC3B II/LC3B I ratio and beclin-1 expression, and inhibited p62 expression. Additionally, it could elevate inflammatory factor level and induce IKK and IκB phosphorylation as well as NF-κB proteins. In in vivo experiments, Bupivacaine inhibited tumor volume and tumor, as well as NF-κB expression. In short, bupivacaine is available to inhibit CRC proliferation through regulating NF-κB signaling pathway, promote apoptosis and autophagy, and can be used as a potential drug to treat CRC in the future.

## Graphical Summary

**Figure:**
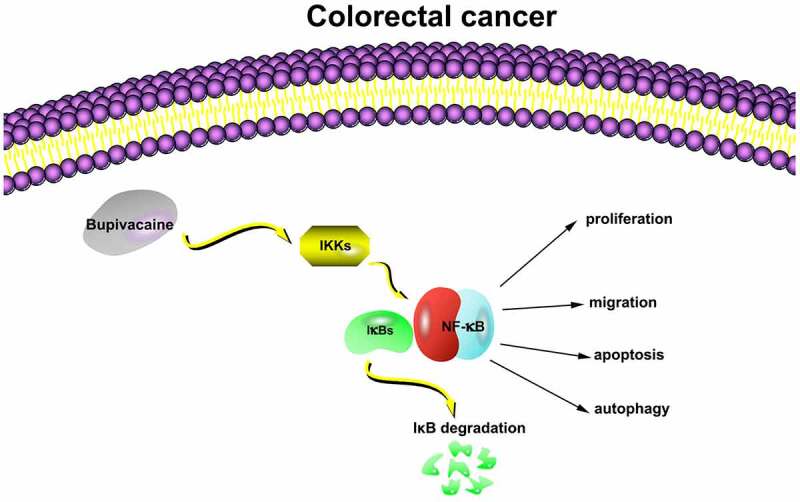

## 1 Introduction

Colorectal cancer (CRC), a universal gastrointestinal tumor, has become the fourth most universal reason for cancer death worldwide [1]. At present, the treatment mainly depends on surgical excision combined with chemotherapy and radiotherapy, but for patients with advanced CRC, survival rate is difficult to improve [2,3]. The surgical efficacy is usually reduced by cancer at high risks of recurrence [4]. The previous studies have reported that the type of anesthetics applied during surgical excision links with a long-term prognosis of cancer [5,6]. Additionally, researchers recently have revealed that an inhaled anesthetic called sevoflurane inhibits CRC migration as well as invasion through regulating miR-203/Extracellular regulatory protein kinase/human matrix metalloproteinase 9 pathway. Based on retrospective research, local anesthesia is available to reduce cancer cell metastasis along with recurrence in patients undergoing surgery [7,8], indicating that anesthetics may act pivotally in cancer development and poor prognosis.

Bupivacaine is a local anesthetic connected to acylamide, commonly applied in surgically removing tumors [9]. Previous studies have revealed that Bupivacaine is available to regulate ras homologous family member A/Rho-related kinase/myosin light chain pathway to inhibit gastric cancer migration [10]. Additionally, studies have reported that Bupivacaine has shown direct anti-cancer impact through activating phosphorylation of Glycogen synthase kinase-3 beta pathway. Recently, researchers found that Bupivacaine inhibited CRC proliferation and migration, but without adverse effects on patients undergoing CRC surgery [11,12].

However, although some progress has been made in the research of Bupivacaine inhibiting cancer, its potential molecular mechanism as a local anesthetic affecting cancer cell biology have not yet been fully identified. This research was to probe into the impact of Bupivacaine on human CRC cell proliferation, apoptosis as well as autophagy, and highlighting its impact on nuclear factor kappa B (NF-κB) signaling pathway in CRC cell.

## 2 Methods

### 2.1 Cell culture

Our team purchased human CRC cells (SW480 & SW620) and human normal colorectal epithelial cell line (FHC) from CAS (China) cell bank, cultured cells in RPMI-1640 mediums (Sigma Aldrich, USA), added 10% newborn calf serum to the medium (HyClone, New Zealand), 1% L-glutamine as well as 1% penicillin-streptomycin (Sigma Aldrich), kept cells in air jacket incubator (Triple Red, UK) with 5% CO_2_, at 37°C, cultured all cells in 24-well plates, applied in experiments when cell fusion reached 70–80%, treated the cells with 1 µM-1 mM Bupivacaine for further experiments after 24 or 48 hours.

#### 2.2 3-(4,5-dimethylthiazol-2-yl)-2 2,5-diphenyl-2 H-tetrazolium bromide (MTT) assay for detecting cell viability

In accordance with the instructions from manufacturer, we applied MTT assay kit (Sangon Biotech, China) to detect cell proliferation, seeded the cells into 96-well plate, cultured them with 1 µM-1 mM Bupivacaine in 24 or 48 h, added 0.5 mg/mL MTT, incubated them for 4 h, removed the supernate, added DMSO, and measured the absorbance at 570 nm through enzyme-labeled instrument.

### 2.3 Flow cytometry for detecting cell apoptosis

The cell apoptosis was detected as described previously [13]. In accordance with the instructions from manufacturer, we collected cells and dyed them with fluorescein isothiocyanate-annexin V and propylene iodide through AV-FITC/PI apoptosis detection kit (Keygen, China). Our team measured AV/PI-positive cells via flow cytometry.

### 2.4 Cell scratch assay

Cultured the cells in a 60 mm culture plate to form a single layer of convergence, later scratched the single layer with a 1 mL of pipettes tip and washed twice with medium. Before taking each image, we made a mark at the bottom of the plate to be sure that all images were taken in the same location, where our team taken a second image 24 h after another occurrence with or without Bupivacaine at different concentrations. Based on these images, we compared the wound healing through Image-Pro Plus software (Media Cyber nets in the United States).

### 2.5 Immunofluorescence

Fixed SW480 and SW620 cells in 4% polyformaldehyde, then sealed them in PBST (Sigma Aldrich) with donkey serum for 1 h before inoculation with the primary antibody: NF-κB (3039, 1:1000, Cell Signaling Technology) overnight in PBST at 4°C, adding FITC or rhodamine-coupled secondary resistance (1:400, Millipore, UK). Later our group re-dyed the slides with 4’, 6-diamidino-2-phenylindole (DAPI Dye) as well as examined them through Olympus BX4 microscope (Watford, UK). At last, we quantified immunofluorescence using ImageJ (National Institutes of Health, Maryland).

### 2.6 Enzyme-linked immunosorbent assay (ELISA)

In accordance with the instructions from manufacturer, our team measured interleukin 1β (IL-1β), Tumor Necrosis Factor α (TNF-α) and Interleukin 6 (IL-6) levels through ELISA kits (BD Biosciences, USA) in market.

### 2.7 Real-time fluorescent quantitative PCR (qRT-PCR)

In accordance with the instructions from manufacturer, our team extracted the total RNA from the cell using TRIzol reagent (Invitrogen), the RNA samples were processed with DNase, and the purity and concentration of total RNA were determined using an ultraviolet spectrophotometer (Eppendorf, Hamburg, Germany). transferred RNA as complementary DNA (cDNA) through PrimeScript reverse transcriptase kits (Takala City, Liaoning Province, China), performed qRT-PCR on the ABI StepOnePlus™ Real-Time PCR System (American Apple Biosystems) through SYBR Green Mix, applied glyceraldehyde-3-phosphate dehydrogenase (GAPDH) as an internal references, and represented quantitative values through 2-ΔΔCt. The primer sequences are listed in Table 1.

**Table 1.** qRT-PCR primer sequence

	Primer sequence (5’3’)
GAPDH	Forward: 5’-CCTCGTCTCATAGACAAGATGGT-3’
	Reserse: 5’-GGGTAGAGTCATACTGGAACATG-3’
Bcl-2	Forward: 5’- CTGGTGGACAACATCGCTCTG −3’
	Reserse: 5’- GGTCTGCTGACCTCACTTGTG −3’
Bax	Forward: 5’- GGATCGAGCAGAGAGGATGG −3’
	Reserse: 5’- TGGTGAGTGAGGCAGTGAGG −3’
Caspase-3	Forward: 5’- GGATCGAGCAGAGAGGATGG −3’
	Reserse: 5’- TGGTGAGTGAGGCAGTGAGG −3’

### 2.8 Western blot

Western blot was performed as previously described [14]. Extracted the total protein from cells via RIPA lysis buffer (Beyotime, China) and measured protein concentration via BCA protein assay kit (Thermo Fisher, USA), separated the protein samples on sodium dodecyl sulfate-polyacrylamide gel electrophoresis (SDS-PAGE) as well as transferred them to polyvinylidene difluoride (PVDF; EMD, Millipore) containing 5% skimmed milk. Later incubated PVDF at 4°C with primary antibody NF-κB (8248, 1:1000) inhibitor of NF-κB (IκB, 9242, 1:1000), phosphorylated IκB (p-IKB, 2859, 1:1000), IκB kinase (IKK, 2682, 1: 1000), phosphorylated IKK (p-IKK, 2697, 1:1000), microtubule-associated protein light chain 3B (LC3B, 2775, 1:1000), Beclin-1 (3495, 1:1000), p62 (5114, 1:1000) from Cell Signaling Technology as well as anti-GAPDH (ab8245, 1:1000) from Abcam. Later, we put them and horseradish peroxidase-coupled-secondary antibody LgG H&L (HRP, 1:2000, ab6721, Abcam) together, applied Western Lightning Plus-ECL to visualize protein band and LAS-3000 Luminescent Image Analyzer (Fujifilm) for band analysis.

### 2.9 Tumor xenograft

The xenotransplantation experiment was performed as described previously [15]. For animal research, our team obtained 24 male Specific Pathogen Free-grade BALB/c nude mice from the The Second Children and Women’s Healthcare of Jinan City Animal Center and put them in conventional facilities, carried out all animal research in accordance with the guidelines of the Animal Care and Use Committee of the Second Children and Women’s Healthcare of Jinan City Hospital and has been approved by the Animal Care and Use Committee,, as well as allocated the animals into 4 groups: Control I Group, Model I Group, Control II Group, and Model II Group. We injected SW480 cells (1.5 × 106) into Control I and model I groups through subcutaneous injection, and SW620 cells (1.5 × 106) into Control II and Model II groups through subcutaneous injection Subsequently, our team injected 0.1 mL of physiological saline into Control I and Control II groups, and injected the same volume of 4.3 mM and 8.6 mM Bupivacaine into Model I and Model II groups, respectively, once a day for 5 times. We weekly recorded the radius (a) and diameter (b) of tumor with vernier caliper. as well as calculated its volume. The calculation of tumor volume: V = (long diameter) × (wide diameter) 2/2. After 4 weeks, our group euthanasia mice with sodium barbiturate (100 mg/kg, catalog number: P3761, Sigma Aldridge, St. Louis, Missouri, U.S.), anatomized the tumor, and assessed its weight with a balance.

### 2.10 Data analysis

Our work expressed the experimental results as mean ± standard deviation, applied SPSS 22 software for data analysis, combined with Student’s *T* test detection and one-way analysis of variance (ANOVA), applied Tukey’s test to make multiple variance corrections to the sample, and significant differences existed in experimental groups when P < 0.05.

## 3 Results

### 3.1 Bupivacaine inhibited CRC cell proliferation

**Figure 1.**
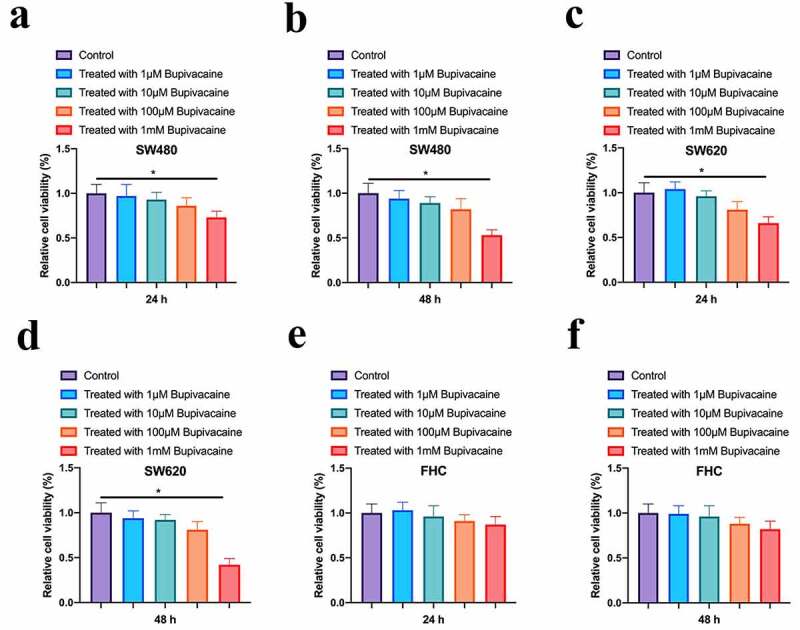
Bupivacaine Inhibited CRC Cell Proliferation

Note: A & B: MTT Assay For Checking SW480 Cell Activity After Receiving 1 μm-1 mm Bupivacaine In 24 And 48 h; C & D:MTT Assay For Checking SW620 Cell Activity After Receiving 1 μm-1 mm Bupivacaine In 24 And 48 h; E & F:MTT Assay For Checking FHC Cell Activity After Receiving 1 μm-1 mm Bupivacaine In 24 And 48 h; Data Shown In Mean ± SD (N = 3); One-Way ANOVA For Calculating The Significance Of Each Group; Applying The Tukey Test For Correcting The Variance. *P< 0.05.

To probe into the impact of Bupivacaine on CRC cell vitality, our team treated CRC cells for 24 or 48 hours with 1 µMm-1 mM Bupivacaine. As shown in Figure 1a-1d, 1 mM Bupivacaine visually inhibited SW480 and SW620 cell vitality. Cell vitality after receiving Bupivacaine in 48 h was lower than than that after receiving Bupivacaine in 24 h. However, 1 mM Bupivacaine had no significant impact on FHC cell vitality in normal CRC cell line (Figure 1e-f). This suggests that Bupivacaine is available to inhibit CRC proliferation.

### 3.2 Bupivacaine inhibited CRC cell migration

**Figure 2.**
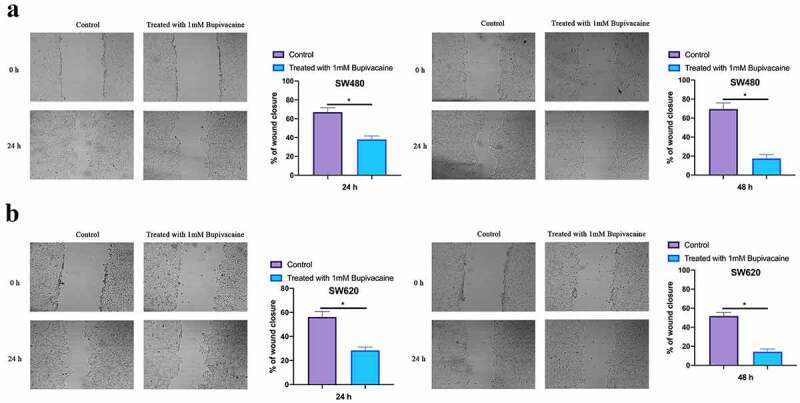
Bupivacaine Inhibited CRC Cell Migration

Note: A & B: Cell Scratch Test For Checking The Cell Migration Capacity In In SW480 And SW620 Cells After Receiving 1 mm Bupivacaine In 24 And 48 h; Data Shown In Mean ± SD (N = 3); One-Way ANOVA For Calculating The Significance Of Each Group; Applying The Tukey Test For Correcting The Variance. *P< 0.05.

Examined the influence of 1 mM Bupivacaine on CRC cells migration through scratch assay. As shown in Figure 2a-2d, SW480 and SW620 cell migration were signally reduced after after receiving 1 mM Bupivacaine in 24 or 48 h in comparison with those in control group. This implies that Bupivacaine is available to inhibit CRC migration.

### 3.3 Bupivacaine promoted CRC cell apoptosis

**Figure 3.**
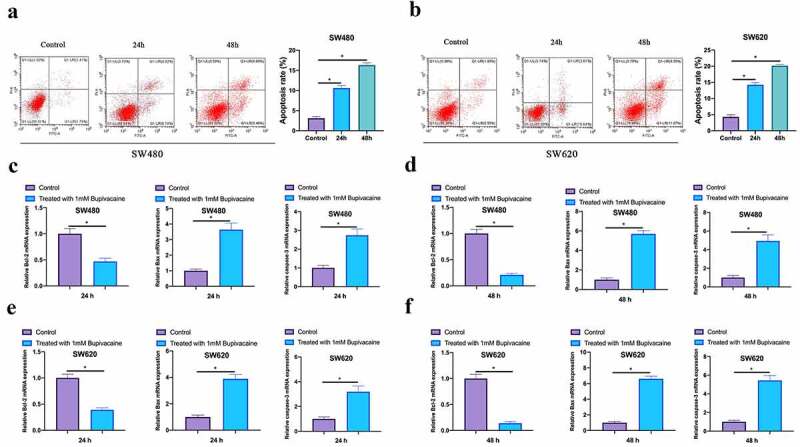
Bupivacaine Promoted CRC Cell Apoptosis

Note: A & B: Flow Cytometry For Checking The Cell Apoptosis Rete In In SW480 And SW620 Cells After Receiving 1 mm Bupivacaine In 24 And 48 h; C & D:Qrt-PCR For Checking Bcl-2, Bax And Caspase-3 Mrna Expression Levels In SW480 And SW620 Cells After Receiving 1 mm Bupivacaine In 24 h And 48 h; Data Shown In Mean ± SD (N = 3); One-Way ANOVA For Calculating The Significance Of Each Group; Applying The Tukey Test For Correcting The Variance. *P< 0.05.

Examined the influence of 1 mM Bupivacaine on CRC cells apoptosis through flow cytometry. As shown in Figure 3a-3b, a significant increase in apoptosis rate of SW480 and SW620 cells after receiving Bupivacaine in 24 or 48 h, featuring time dependency. Additionally, Bupivacaine inhibited Bcl-2 expression in SW480 and SW620 cells, promoting caspase-3 and Bax expressions (3 C-3 F). This suggests that Bupivacaine is available to signally promote CRC cell apoptosis.

### 3.4 Bupivacaine promoted CRC cell autophagy

**Figure 4.**
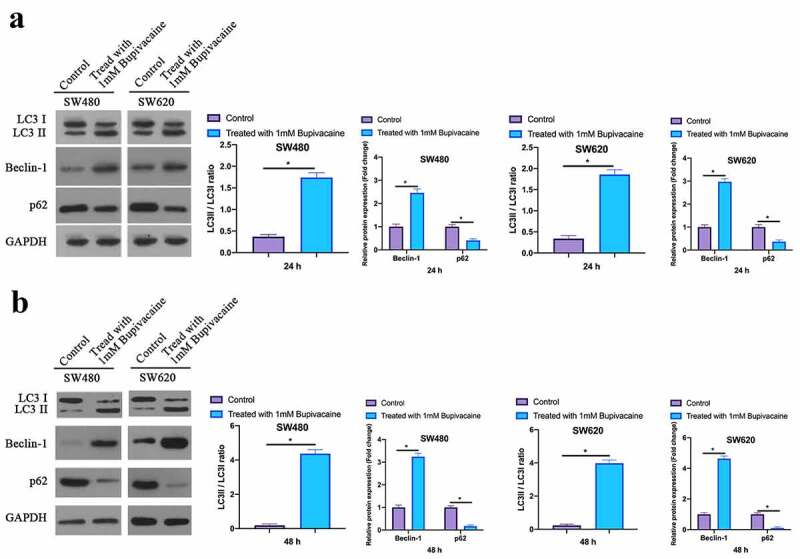
Bupivacaine Promoted CRC Cell Autophagy

Note: A: Western Blot For Checking LC3B, Beclin-1 And P62 Protein Expression Levels In SW480 And SW620 Cells After Receiving 1 mm Bupivacaine In 24 h; B: Western Blot For Checking LC3B, Beclin-1 And P62 Protein Expression Levels In SW480 And SW620 Cells After Receiving 1 mm Bupivacaine In 48 h; Data Shown In Mean ± SD (N = 3); One-Way ANOVA For Calculating The Significance Of Each Group; Applying The Tukey Test For Correcting The Variance. *P< 0.05.

Examined the influence of receiving 1 mM Bupivacaine in 24 or 48 h on autophagy-related protein expression in CRC through Western blot. After treament, Beclin-1 expression and LC3B II/LC3B I ratio were elevated signally while p62 expression was decreased in SW480 and SW620 cells (Figure 4a-4d). It suggested that Bupivacaine is available to signally promote CRC cell autophagy.

### 3.5 Bupivacaine inhibited NF-κB activation in CRC cells

**Figure 5.**
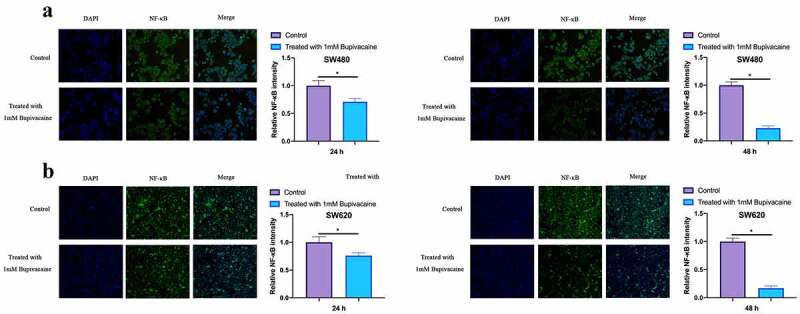
Bupivacaine Inhibited NF-Κb Activation In CRC Cells

Note: A & B: Immunofluorescence For Checking NF-Κb Activation In SW480 Cells After Receiving 1 mm Bupivacaine In 24 h Or 48 h; C & D: Immunofluorescence For Checking NF-Κb Activation In SW620 Cells After Receiving 1 mm Bupivacaine In 24 h And 48 h; Data Shown In Mean ±SD (N = 3); One-Way ANOVA For Calculating The Significance Of Each Group; Applying The Tukey Test For Correcting The Variance. *P< 0.05.

Our work examined the influence of receiving 1 mM Bupivacaine on NF-κB activation in CRC cells through immunofluorescence (IF). As shown in Figure 5a-5d, after treating SW480 and SW620 cells with Bupivacaine in 24 or 48 h, active NF-κB fluorescence intensity was signally reduced with time dependence. This suggests that Bupivacaine is available to signally inhibit CRC cell activation.

### 3.6 Bupivacaine inhibited CRC inflammation factors

**Figure 6.**
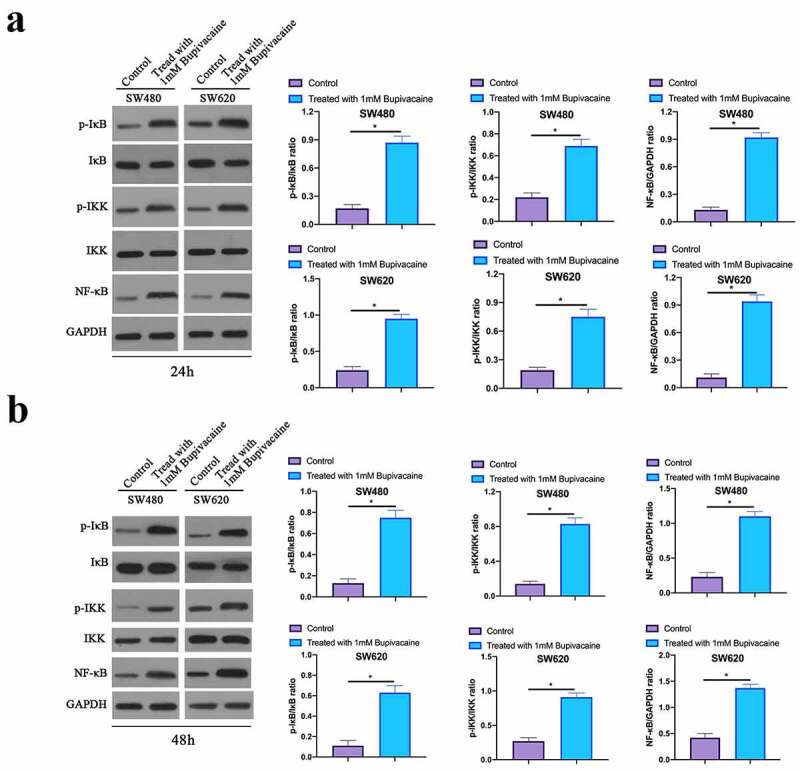
Bupivacaine Inhibited CRC Inflammation Factors

Note: A & B: ELISA For Checking TNF-Α, IL-1β, And IL-6 Levels In SW480 And SW620 Cells After Receiving 1 mm Bupivacaine In 24 h And 48 h; One-Way ANOVA For Calculating The Significance Of Each Group; Applying The Tukey Test For Correcting The Variance. *P< 0.05.

Examined the influence of receiving 1 mM Bupivacaine in 24 or 48 h on inflammation factors of carcinoma of colon and rectum through ELISA. As shown in Figure 6a and 6b, after treating SW480 and SW620 cells with 1 mM Bupivacaine in 24 or 48 h, IL-1 beta, IL-6, and TNF-α levels were visually reduced. This suggests that Bupivacaine is available to signally inhibit CRC inflammation.

### 3. 7 Bupivacaine inhibited CRC NF-κB signaling pathway

**Figure 7.**
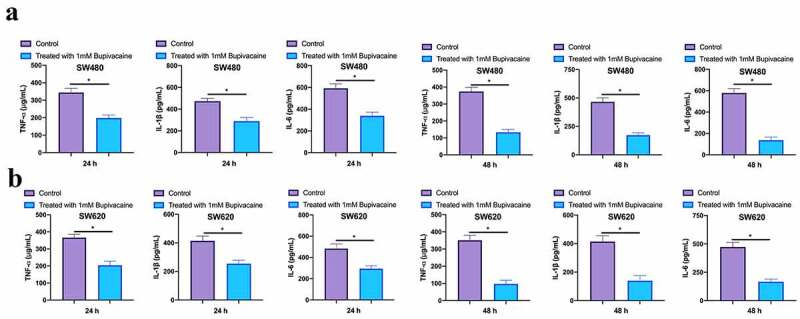
Bupivacaine Inhibited CRC NF-Κb Signaling Path

Note: A & B: Western Blot For Checking NF-Κb, Iκb Phosphorylation And IKK Phosphorylation Protein Expression In SW480 And SW620 Cells After Receiving 1 mm Bupivacaine; Data Shown In Mean ± SD (N = 3); One-Way ANOVA For Calculating The Significance Of Each Group; Applying The Tukey Test For Correcting The Variance. *P< 0.05.

Next, our work examined the influence of receiving 1 mM Bupivacaine in 24 or 48 h on CRC NF-κB signaling pathway through western blot. As shown in Figure 7a and 7b, Bupivacaine significantly reduced NF-κB, phosphorylation IKK and IκB levels in SW480 and SW620 cells. This suggests that Bupivacaine is available to signally inhibit CRC NF-κB signaling pathway.

### 3. 8 Bupivacaine inhibited tumor growth in the body

**Figure 8.**
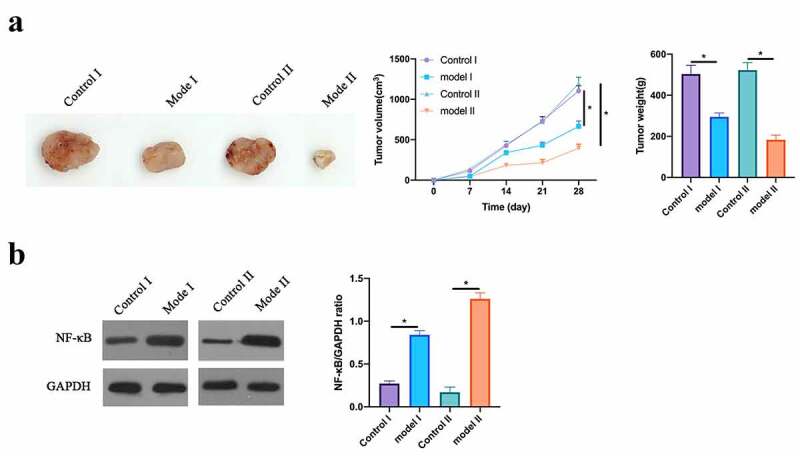
Bupivacaine Inhibited Tumor Growth In Body

Note: A: Photos, Volume And Weight Of Mouse Tumors After Receiving 4.3 mm And 8.6 mm Bupivacaine; B: Western Blot For Detecting NF-Κb Expression After Receiving 4.3 mm And 8.6 mm Bupivacaine; Data Shown In Mean ± SD (N = 6). One-Way ANOVA For Calculating The Significance Of Each Group; Applying The Tukey Test For Correcting The Variance. *P< 0.05。

To further prove our findings, our work conducted *in vivo* experiments. In order to determine the optimal dose, 5 different doses of bupivacaine (2.15, 4.30, 6.45, 8.60, 10.75 mM) were injected into the nude mice implanted with tumors, and it was found that 4.3 mM began to have inhibitory effect on tumor and 8.6 mM began to obtain the maximum inhibitory effect (results not shown), so 4.3 mM and 8.6 mM bupivacaine were chosen for administration. Based on the findings, 4.3 mM and 8.6 mM Bupivacaine obviously inhibited mice volume and weight (Figure 8a). Additionally, western blot findings revealed that Bupivacaine obviously inhibited NF-κB phosphorylation in CRC in the body (Figure 8b). This suggests that Bupivacaine inhibits rectal cancer growth in the body.

## 4 Discussions

In this research, we found that Bupivacaine signally inhibits CRC cell activity while promoting its apoptosis and autophagy. Further studies have shown that Bupivacaine links with Bcl-2, Bax and caspase-3 gene expressions regulating apoptosis, and with the autophagy-related p62, Beclin-1, and LC3B expression proteins. Additionally, our team have found that Bupivacaine signally inhibits phosphorylation of proteins IKK, IκB and NF-κB relative to NF-κB signaling pathway in cells.

Inducing cancer cell apoptosis is among the vital indicators to evaluate the anti-tumor drugs inhibiting cancer cells growth [16,17]. Studies by Bundscherer A et al. have shown that Bupivacaine inhibits colon cancer cell proliferation, but has no significant impact on colon cancer cell apoptosis. We found that 1 mM Bupivacaine signally inhibits CRC cell apoptosis rate, possibly because the drugs we used was at much higher concentration than that by Bundscherer A et al. Bax is a pro-apoptosis protein, which elevates mitochondrial membrane permeability after receiving apoptosis signal, resulting in decreased membrane potential and promoting the release of apoptosis factors to cytokines. While Bcl-2 in case of mitochondrial membrane damage is available to repair mitochondrial control and reduce mitochondrial membrane permeability, thus playing an anti-apoptosis effect [18,19]. Additionally, cysteine aspartic acid specific protease can inhibit apoptosis [20] when activated. In this work, we found that 1 mM Bupivacaine visually elevated mRNA expression in Bax and caspase-3 while inhibited it in Bcl-2. In addition, ropivacaine can also inhibit the migration of colorectal cancer, which is consistent with previous studies [21].

Cancer cell autophagy is a process of degrading damaged cell organs or proteins in the eukaryotic cells. Amidst preventing and treating cancer, the search for reliable cancer cell autophagy regulators has become a heated topic for scientists [22,23]. Beclin-1, p62, and LC3B are key proteins involved in cell autophagy regulation. Beclin-1 is responsible for collecting autophagy protein and initiating autophagy [24]. As for p62 protein, a suitable protein between autophagy and substrate, acts as a molecular regulator during autophagy. LC3 forms LC3B I through participating in ubiquitination, which is later lipidized into LC3B II. LC3B II/LC3B I ratio can be regarded as a marker of autophagy degradation [25]. Previously published research has shown that anesthetics act actively in regulating cell autophagy [26]. In this research, we found that Bupivacaine observably elevated LC3B II/LC3B I ration and Beclin-1 expression while inhibited p62 expression.

A great many studies have revealed that activating NF-B signaling pathway acts pivotally in the process of CRC cell proliferation, apoptosis, angiogenesis as well as metastasis [27,28]. During activating NF-κB, TLR4 receptor binding to ligand results in activating IKK complex, while IKK activation further promotes IκB phosphorylation, which then triggers the release of NF-κB from nucleus and further stimulates the release of inflammatory factors [29]. In CRC occurrence, the role of inflammation in tumor microenvironment is unquestionable. Immunotherapy for NF-κB has great potential in improving the survival rate of CRC patients. Studies have implied that Bupivacaine acts actively in treating diseases through inhibiting the NF-κB signaling pathway activation. We found that Bupivacaine visually inhibited IKK phosphorylation, IκB phosphorylation and NF-κB expression in CRC cells, a process that may have beneficial impact on inhibiting CRC proliferation while promoting apoptosis and autophagy.

## 5 Conclusion

To sum up, our findings prove that Bupivacaine inhibits CRC proliferation through blocking the NF-κB signaling pathway activation, promoting apoptosis and autophagy, thus providing strong data support for Bupivacaine as a drug for CRC in the future.

## Abbreviations

Nuclear factor κBNF-κB

colorectal cancerCRC

**3-(4,5-dimethylthiazol-2-yl)-2 2,5-diphenyl-2H-tetrazolium bromide**MTT

Interleukin 1βIL-1β

Tumor Necrosis Factor αTNF-α

Interleukin 6IL-6

Real-time fluorescence quantitative PCRqRT-PCR

Glyceraldehyde-3-phosphate dehydrogenaseGAPDH

inhibitor of NF-κBIκB

Phosphorylated IκBp-IκB

IκB kinaseIKK

Phosphorylated IKKp-IKK

Microtubule-associated protein light chain 3BLC3B
